# Research Progress, Application, and Industrialization Prospects of Circular RNA Vaccines in Viral Diseases

**DOI:** 10.3390/vaccines14090781

**Published:** 2026-09-07

**Authors:** Dongjie Cai, Xingling Li, Ruoxu Wang, Chen Lin, Jing Wen, Bin Tian

**Affiliations:** 1 College of Veterinary Medicine, Sichuan Agricultural University, Chengdu 611130, China; 2 Sichuan Key Laboratory of Agricultural Animal Diseases and Veterinary Public Health, Chengdu 611130, China

**Keywords:** RNA vaccines, circular RNA vaccines, messenger RNA, self-amplifying RNA, lipid nanoparticles, circularization strategies, immune response, targeted delivery

## Abstract

RNA vaccines—comprising linear mRNA, self-amplifying RNA, and circular RNA (circRNA)—constitute a core next-generation platform for the prevention and control of viral diseases; among these, circRNA vaccines possess notable structural stability, yet their technical bottlenecks and application prospects in veterinary medicine have not been systematically reviewed. This review synthesizes current research on circRNA vaccine design, circularization strategies, translation mechanisms, delivery systems, and immunological outcomes, and compares their antiviral performance with that of linear mRNA vaccines. Owing to their covalently closed circular conformation, circRNA vaccines exhibit enhanced resistance to nucleases and superior thermal stability, enabling sustained transfection activity at ambient temperatures without reliance on strict cold chains; through cap-independent translation driven by internal ribosome entry sites or N^6^-methyladenosine modifications, and in conjunction with optimized circularization protocols and lipid nanoparticle carriers, circRNA vaccines elicit substantially higher antiviral IgG titers and durable antigen-specific T-cell memory relative to linear mRNA vaccines. These vaccines have been deployed against COVID-19, monkeypox, influenza, and livestock viral diseases, demonstrating strong adaptability to viral variants and compatibility with mucosal or needle-free administration routes. CircRNA vaccines are well suited for both emergency outbreak response and routine immunization programs; nevertheless, challenges persist, including low circularization efficiency for long sequences, elevated manufacturing costs, and inadequate quality control standards. Addressing these issues through improved production workflows and delivery technologies adapted to resource-limited settings will be critical to establishing circRNA vaccines as a pillar of livestock disease management and as a strategic reserve for emerging zoonotic threats.

## 1. Introduction

RNA vaccines, as an important branch of nucleic acid vaccines, have achieved a leap from basic research to large-scale industrialization during the COVID-19 pandemic, demonstrating multiple advantages including rapid design, flexible iteration, cell-free large-scale manufacturing, and the ability to induce both humoral and cellular immunity [1,2,3,4]. Conventional vaccines have yielded considerable success in routine infectious disease control, yet they struggle to address challenges posed by rapidly mutating pathogens and sudden outbreaks. In this context, the “plug-and-play” (i.e., antigen sequences can be directly swapped without re-engineering the core circRNA backbone and production process) platform property of RNA vaccines delivers unique strategic value: antigen constructs can be generated within hours after target sequence release, and a unified production workflow can be adapted to diverse antigens, an advantage fully validated during the global deployment of COVID-19 vaccines [1,3,4,5]. Nevertheless, linear messenger RNA (mRNA) is inherently sensitive to nucleases and dependent on cold-chain logistics. Despite the safety and efficacy improvements brought on by nucleoside modification and lipid nanoparticle (LNP) delivery technologies, unresolved limitations remain, including short in vivo half-life, insufficient thermal stability, and a relatively high incidence of certain systemic adverse reactions [2,3,4,6,7,8]. These core bottlenecks have driven continuous exploration of next-generation RNA vaccine platforms [3,4,6,7].

To date, self-amplifying RNA (saRNA) and circular RNA (circRNA) have emerged as two major developmental directions. saRNA reduces the required administration dosage via intracellular self-amplification, but its longer sequence increases manufacturing complexity and process difficulty [3,4,9]. In contrast, circRNA features a covalently closed circular structure that confers stronger nuclease resistance and a significantly prolonged half-life compared with linear mRNA, enabling more sustained antigen expression in vivo [1,3,4,6]. It initiates translation through an internal ribosome entry site (IRES) or N^6^-methyladenosine (m^6^A) modification, which can reduce or even eliminate the demand for nucleoside modification and simplify production processes; it also maintains stable transfection efficiency after two weeks of storage at room temperature, showing prominent advantages in thermal stability [3,4,6,8]. Nevertheless, several diverging viewpoints and unresolved controversies remain in the field: the intrinsic innate immunogenicity of circRNA is still debated, with inconsistent conclusions across different research systems; mainstream circularization strategies (including permuted intron–exon systems and enzymatic ligation) differ greatly in production yield, impurity profiles and industrial scalability, with no consensus on a universal large-scale technical route; and the tissue-dependent activity of IRES elements also leads to heterogeneous translation efficiency across different application scenarios. Recently, integrating self-replicating elements with circRNA backbones has emerged as a promising exploratory direction, but relevant studies are still in the preliminary stage.

The primary focus of this review is circRNA vaccine platforms. We take linear mRNA vaccines and self-amplifying RNA (saRNA) vaccines as control technical routes for comparative analysis and systematically summarize the core strategies of target-oriented circRNA vaccine design, key technological breakthroughs, unresolved bottlenecks and translational prospects. On the whole, self-replicating circRNA exhibits comprehensive advantages in immunogenicity, thermal stability and production cost control, and is regarded as a highly promising next-generation RNA vaccine technology with prominent industrialization potential; however, long-sequence circularization efficiency, standardized quality control systems and delivery system adaptability remain major bottlenecks restricting its clinical and industrial translation. This review aims to provide a reference for the research, development and translational application of next-generation RNA vaccines.

## 2. Overview of RNA Vaccine Technology

### 2.1. Technical Characteristics of mRNA Vaccines

Linear mRNA vaccines are non-replicating RNA. Their typical structure consists of a 5′ cap structure, a 5′ untranslated region (5′-UTR), a viral antigen open reading frame (ORF), a 3′-UTR, and a polyadenosine tail (poly(A)) [3,4,10]. Their sequence modularity is high, allowing rapid replacement of antigen genes from different viruses to meet the prevention and control needs of various viral diseases. Linear mRNA has poor thermal stability [11] and is highly dependent on cold-chain storage, transport and delivery systems [12]; its in vivo half-life is short, and the duration of antigen expression is limited [13], usually requiring multiple immunizations; and its adaptability to viral variants is insufficient, making it difficult to cope with rapidly mutating emerging and suddenly occurring viral diseases [14].

### 2.2. Technical Characteristics of saRNA Vaccines

Self-amplifying RNA is replicating RNA containing viral replication-related elements, an antigen-coding sequence, and an in vitro transcription (IVT) backbone [15]. The backbone is mostly engineered from alphavirus genomes [16], with a full length of approximately 9–12 kb. In addition to the basic elements of linear mRNA, it also carries the coding region for non-structural proteins 1–4 (nsP1-nsP4) and a subgenomic promoter, enabling self-amplification within host cells without producing infectious viral particles [17], thus offering a relatively high safety profile. After entering the cytoplasm, saRNA translates nsP1-nsP4, which assemble into an RNA-dependent RNA polymerase (RdRP) that initiates intracellular self-amplification [18], generating large quantities of subgenomic RNA-encoding viral antigens, achieving efficient and sustained antigen expression, significantly enhancing immunogenicity, and inducing potent antiviral immunity at low doses [19]. saRNA has a long sequence and a complex structure, leading to complicated synthesis processes and high large-scale production costs [17]; in the presence of co-infecting viruses, the saRNA of the alphavirus replicon can recombine with viral RNA to produce novel or altered virus strains. Meanwhile, due to the cytotoxic effects induced by its replication mechanism, saRNA leads to cell damage and impaired host cell viability [20].

### 2.3. Structure and Biosynthesis of circRNA

circRNA is a covalently closed circular single-stranded RNA (ssRNA) [21] formed through back-splicing, in which a downstream 5′ splice donor site is covalently linked to an upstream 3′ splice acceptor site, resulting in no free 5′ cap or 3′ poly(A) tail, thus making it more stable than linear RNA and less susceptible to degradation by exonucleases [22]. Similar to mRNA, natural circRNA contains non-coding regions and protein-coding regions [23]. Based on sequence origin, it can be classified into exonic, intronic, and exon-intron hybrid types [24]. In eukaryotes, circRNA is mainly formed through back-splicing, where a downstream donor site is cis-paired with an upstream acceptor site. In the mainstream back-splicing model, exon circularization occurs directly from the nascent linear transcript, with flanking introns being spliced in a head-to-tail manner and the intermediate region being excised to form an RNA circle [25]. In addition to back-splicing, endogenous circRNA can also be generated through direct circularization of intron sequences [26]. Currently, the main method for in vitro synthesis of circRNA is to ligate the ends of linear RNA precursors to form a covalently closed circle. Two approaches are employed: chemical synthesis and enzymatic strategies. An advantage of chemical synthesis of linear RNA is that a 5′ monophosphate can be directly introduced during synthesis to facilitate future circularization [27]. However, due to high purification costs and low yields, chemical synthesis can only produce RNA with lengths of fewer than 50 to 70 nucleotides. Enzymatic strategies are typically achieved through IVT reactions, which include a DNA template, reaction buffer, and bacteriophage RNA polymerase [25]. The best-practice quality control standards for circRNA research cover six modules [28]: purification and expression profiling, circRNA validation and quantification, in situ hybridization detection, circRNA knockdown, circRNA overexpression, and regulatory mechanism investigation. Each module defines corresponding operational specifications and quality control rationales, covering the complete experimental workflow from sample preparation and bioinformatic analysis to functional validation, and provides a unified quality evaluation basis for downstream applications such as circRNA vaccine development.

### 2.4. Functions of Endogenous Non-Coding circRNAs

Studies have found that the endogenous non-coding circRNA SMARCA5 can bind to host gene loci to form R-loops, leading to premature transcriptional termination of SMARCA5 at exon 15, downregulating full-length gene expression and producing truncated, non-functional isoforms; meanwhile, an overexpression of circSMARCA5 can significantly enhance the sensitivity of breast cancer cells to cytotoxic drugs [29]. Research has confirmed that endogenous non-coding circRNAs exert biological functions by acting as a competing endogenous RNA (ceRNA) [30]. Endogenous non-coding circRNAs can serve as a miRNA sponge involved in post-transcriptional gene regulation, functioning as a ceRNA by blocking miRNA-mediated regulation of target genes. Lacking a 5′ cap and 3′ tail, endogenous non-coding circRNAs can only complete translation through cap-independent mechanisms. The main translation mechanisms are m^6^A-mediated and IRES-mediated translation. m^6^A is an important RNA modification that regulates RNA splicing, stability, and translation, although the regulatory mechanisms of m^6^A on endogenous non-coding circRNA biogenesis and function have not yet been fully elucidated. Studies have found that the endogenous non-coding circRNA ZNF609 possesses translation potential [31]. However, the regulatory mechanisms of endogenous non-coding circRNA translation, as well as the processes of translation elongation and termination, have still not been fully clarified. Zhou et al. summarized three modes of circRNA–protein interaction: a single endogenous non-coding circRNA simultaneously binds two proteins and promotes their interaction; endogenous non-coding circRNA binds protein A and regulates the interaction between protein A and protein B; and endogenous non-coding circRNA binds two proteins that have already formed a complex and dissociates their interaction, thereby forming a multi-component complex and regulating downstream signals [30].

### 2.5. Vaccine-Compatible Properties of circRNA

circRNA is widely present in eukaryotic cells and viral genomes and represents a potential vaccine adjuvant with unique advantages (Table 1). Studies have shown [32] that when exogenous circRNA is co-delivered with soluble protein, it can effectively induce antigen-specific antibody production and T-cell activation. It can directly activate the RNA pattern recognition receptor RIG-I, promoting the filamentation of the adaptor protein MAVS and the activation of downstream transcription factor IRF3, thereby enhancing the intensity and duration of the immune response and providing key support for improving vaccine efficacy. At the same time, numerous studies have demonstrated the advantages of circRNA in activating innate immune cells and dendritic cells and delivering encoded protein antigens [33,34]. Furthermore, preclinical studies in cellular and animal models have demonstrated that circRNA exhibits higher translation durability compared with linear RNA [35]. Chen, Robert et al., by optimizing in vitro synthesized circular mRNA, demonstrated that engineered circRNA can produce more protein than mRNA in vitro and exhibits higher translation durability both in vitro and in vivo. In addition, re-administration of circRNA after a two-week interval showed no significant decrease in expression, supporting the feasibility of repeated use of circRNA in the same subject. Notably, the introduction of IRES into vectors for ectopic expression of circRNA or in vitro synthesized circRNA vectors has resulted in efficient protein translation in cells and animals [36]. circRNA does not necessarily require nucleoside modification. Existing preclinical evidence suggests that it may have lower intrinsic innate immune activation than nucleoside-modified linear mRNA, yet relevant safety conclusions remain to be verified in clinical trials [37].

#### 2.5.1. Industrialization Comparison: circRNA and Linear mRNA

From an industrialization perspective, linear mRNA vaccines have established a highly mature and standardized GMP manufacturing workflow with well-established process control systems [38].

Covering core steps including in vitro transcription and multi-stage purification, all critical procedures have clear yield and purity acceptance criteria, forming a widely recognized industrial production and cost benchmark [39].

In contrast, circRNA production requires an additional circularization step based on linear precursor synthesis. Current mainstream circularization strategies include chemical synthesis, T4 RNA ligase ligation, the permuted intron–exon (PIE) system based on group I intron self-splicing, and the group II intron self-splicing circularization system, each with respective advantages in applicable sequence length, exogenous sequence residue and immunogenicity. Antigen-encoding sequences, usually longer than 1 kb, are susceptible to RNA secondary structure interference, which significantly increases the difficulty of circularization [40].

After circular RNA is synthesized via in vitro transcription (IVT) coupled with self-splicing or enzymatic circularization, the crude product forms a complex system composed of multiple components: uncyclized linear precursors, intact circular RNA, circular RNA concatemers, and nicked damaged circular RNA [35].

The prevailing purification strategy employs RNase R digestion to deplete linear precursors, followed by multi-step size-exclusion high-performance liquid chromatography (SE-HPLC) to remove nicked circular by-products. However, this combined workflow delivers only limited improvements in purity and yield, and SE-HPLC is plagued by severe peak overlap. Experimental replication of this purification procedure yields a final product purity of only 41% and an overall yield of 45% [41]. Its overall recovery is markedly lower than that of linear mRNA production, accompanied by higher process complexity, rendering it unsuitable for large-scale manufacturing. Accordingly, standardized GMP manufacturing is not yet well established.

Currently, the production cost of circRNA is significantly higher than that of linear mRNA. Linear mRNA benefits from mature technology and gradually decreasing costs, while circRNA production requires specialized circularization technology with a more complex process flow, low circularization efficiency for long sequences and complicated downstream purification workflows. Full commercial mass production of circRNA has not been realized at this stage [5,8]. Although circRNA has long-term cost reduction potential through circularization scaffold optimization, purification workflow simplification and production scale-up, its overall industrial maturity still lags far behind the linear mRNA platform, and large-scale production and application are still restricted by core technical bottlenecks [42,43].

#### 2.5.2. Quality Control Standards and Regulatory Framework

For linear mRNA vaccines, the World Health Organization (WHO) has issued dedicated official technical guidance documents, establishing a systematic regulatory and quality control framework for the assessment of quality, safety and efficacy of prophylactic mRNA vaccines against infectious diseases. This guidance has incorporated key quality attributes including mRNA integrity, purity, capping efficiency, poly(A) tail length and double-stranded RNA (dsRNA) residue into the scope of quality control requirements and clarified full-chain quality control principles spanning from starting materials and in-process manufacturing control to final product release [44].

Circular RNAs present diverse topological structures and complex impurity profiles. There is currently no unified gold standard for their quality control, which necessitates comprehensive characterization via multiple orthogonal techniques. The multi-dimensional analytical system established in a study includes denaturing polyacrylamide gel electrophoresis, RNase R/RNase H digestion assays, mass spectrometry, high-performance liquid chromatography and junction sequencing. These methods enable component separation and quantification, topological structure verification, molecular weight confirmation, purity analysis and sequence fidelity validation, respectively [45]. Through cross-validation across multiple assays, the closed-loop fraction, impurity profile and ligation accuracy of products can be systematically evaluated, providing methodological support for the quality control of circRNA vaccines.

#### 2.5.3. Clinical Translation Progress

(1) Human Vaccines

Circular RNA (circRNA) vaccines remain in the early stage of research and development overall. Existing R&D projects mainly focus on two areas: prophylactic vaccines against COVID-19 and therapeutic vaccines for melanoma. Relevant candidate products have only demonstrated antigen expression efficiency, immunogenicity and disease protective efficacy in animal models such as mice and rhesus macaques, and have not yet entered clinical trials [46].

Messenger RNA (mRNA) vaccines have achieved commercial launch and formal approval as COVID-19 products. Candidate vaccines targeting a variety of infectious diseases, including rabies, seasonal influenza, Zika virus and respiratory syncytial virus, have advanced to different clinical stages from Phase I to Phase IV, with a well-established clinical translation pathway [10].

(2) Veterinary Vaccines

Veterinary mRNA vaccines are generally in the preclinical research and development stage. For a variety of important animal diseases, including foot-and-mouth disease virus, rabies virus, African swine fever virus and porcine reproductive and respiratory syndrome virus, verification of immunogenicity and challenge protection efficacy has been completed in animal models [2]. Early literature scarcely reported veterinary circRNA vaccine candidates; however, several recent pre-clinical studies have been published [10].

Almost all circRNA veterinary vaccines remain in the preclinical research stage. Candidates targeting classical swine fever virus, novel duck reovirus and other pathogens have only completed challenge studies in target animals, and have not entered clinical development [47,48].

**Table 1:** Comparison of core technical characteristics of three types of RNA vaccines.

Vaccine	Cold-Chain Dependence	Stability	Variant Adaptability	Antigen Expression	IgG Relative Titer	Manufacturing	Advantages	Disadvantages	References
Linear mRNA	High dependence	Low	Weak	Transient	1-fold	Simple & mature	Industrial	Cold-chain dependent	[1,6,11,13,16,21,49]
SaRNA	Dependence	Moderate	Moderate	High & prolonged	1.1–1.3-fold	Complex	Self-amplifying	High cost	[17,18,19]
CircRNA	Low dependence (preclinical formulation data)	Relatively high (intrinsic closed-loop property, preclinically validated)	Formidable	Sustained & durable	Approx. 3.8-fold (mouse model, SARS-CoV-2 antigen)	Medium	Thermostable	Low translation	[6,8,21,22,31,34,50]

## 3. Immune Response Mechanism of circRNA Vaccines

**Figure 1:**
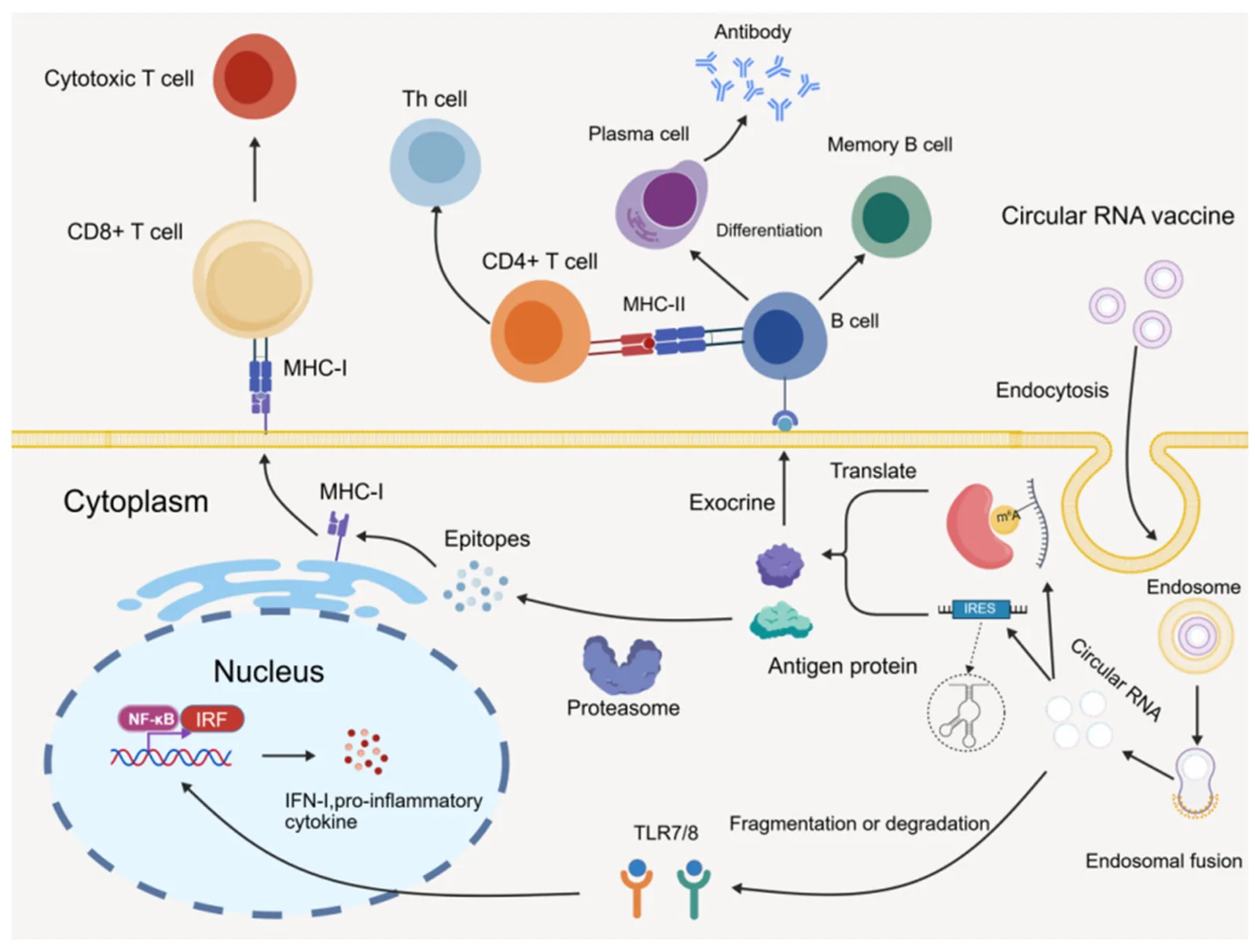
Immunological mechanism of circRNA vaccines.

The immune response of circRNA vaccines follows the core mechanism of “delivery and cellular entry antigen expression and presentation innate immune adjuvant effect adaptive immune initiation and memory formation” (Figure 1). After entering the body, circRNA vaccines mostly use LNPs as the main delivery vehicle [51,52,53,54,55]. Cells take up LNPs through endocytosis and form endosomes; subsequently, in the acidic endosomal environment, LNPs achieve endosomal escape via ionizable lipid-induced membrane damage or the “proton sponge effect”, releasing circRNA into the cytoplasm [51,52,53,54,55].

After entering the cytoplasm, circRNA primarily relies on IRES- or m^6^A-mediated cap-independent translation mechanisms to continuously synthesize the target antigen, and there are significant differences in the mode of competition for translation initiation factors compared with linear mRNA [56,57]. Non-secreted antigens produced by translation are degraded into short peptide fragments by the proteasome. Among them, the immunoproteasome specifically expressed in antigen-presenting cells (APCs) can preferentially generate high-quality antigenic peptides of 8–10 amino acids that are more suitable for binding to major histocompatibility complex class I (MHC-I) molecules by altering the structure of the substrate-binding pocket. These peptides are transported into the endoplasmic reticulum lumen by the transporter associated with antigen processing, where they bind to MHC-I molecules to form stable complexes, which are then transported to the cell surface to activate CD8-positive (CD8^+^) T cells to produce cytotoxic effects [46,58,59]. Secreted antigens encoded by circRNA can be directly recognized by B cells or taken up by APCs and presented to CD4-positive (CD4^+^) T cells via the major histocompatibility complex class II (MHC-II) pathway, jointly promoting B cell differentiation, antibody production, and memory B cell formation.

CircRNA itself can be recognized by multiple pattern recognition receptors such as RIG-I, MDA5, PKR, TLR3, and TLR7/8, activating downstream NF-κB and IRF signaling cascades, inducing the expression of type I interferons and pro-inflammatory cytokines, upregulating the levels of costimulatory molecules such as CD80 and CD86 on the surface of APCs, and promoting APC maturation and enhancing their antigen-presenting capacity [33,34,54]. Compared with long-chain linear mRNA, circRNA generally exhibits a lower level of phosphorylation, exerting a weaker inhibitory effect on host protein translation, which is conducive to maintaining more durable antigen expression [34]. The unique closed-loop structure of circRNA endows it with far greater in vivo stability than linear mRNA, significantly prolonging the antigen exposure window in vivo and providing the basis for the formation of long-lasting immune memory.

## 4. Synthesis Methods of circRNA Vaccines

Four mainstream circularization systems are applied for in vitro synthesis of circRNA vaccines (Table 2): the permuted intron–exon (PIE) system, the T4 RNA ligase 2 (T4 Rnl2)-mediated enzymatic circularization system, the linear RNA self-circularization (LRC) system, and the complete self-splicing intron (CIRC) system.

### 4.1. PIE System

The PIE system relies on the self-splicing principle of group I introns. It requires only guanosine triphosphate and Mg^2+^ to complete the covalent circularization of linear RNA precursors through a two-step transesterification reaction [40], serving as a classic scaffold suitable for the preparation of long RNA fragments both in vitro and in vivo. This system has a wide range of applications and simple reaction conditions [60]; however, the natural system exhibits a significant decrease in circularization efficiency for long viral antigen sequences over 1 kb, and residual exogenous scar sequences may pose a potential risk of immune interference [35,61]. Current optimization efforts mainly focus on four aspects:

(1) Introduction of homology arms and spacer sequence engineering, increasing the circularization efficiency of 5 kb long-chain RNA from 0% to 87%, overcoming the bottleneck of long-fragment circularization [62].
(2) Development of a scarless PIE derivative system based on the *Anabaena* intron, which requires only the “A, T” dinucleotide to maintain the molecular characteristics of circularization, establishing a PIE-derived system completely free of exogenous sequences [60].
(3) Using cryo-electron microscopy to resolve the high-resolution structure of the rearranged T4 bacteriophage thymidylate synthase gene intron (T4Td), establishing a structure-guided circularization scaffold engineering paradigm that greatly enhances the circularization efficiency of the T4td-PIE system [63].
(4) Repurposing natural complete introns to construct circularization technology and directly utilizing natural complete group I/II introns to achieve circularization without artificial splitting and engineering under mild reaction conditions, enabling the scarless circularization of ultra-large 14 kb RNA fragments with extremely low product immunogenicity [64].

The PIE system is the most widely adopted platform for circRNA vaccines, with pre-clinical immunization-challenge validations in mice and rhesus macaques against SARS-CoV-2, monkeypox and other viruses. Nevertheless reduced efficiency for long antigens and potential immune interference from scar sequences restrict its in vivo performance [6,65].

### 4.2. T4 Rnl2 Enzymatic Ligation System

T4 Rnl2 catalyzes the formation of a phosphodiester bond between the 3′ and 5′ ends of RNA through an enzymatic reaction. It can utilize the RNA’s own secondary structure or a complementary DNA (cDNA) splint to achieve precise, scarless circularization, serving as the core scaffold for the standardized preparation of small- to medium-sized circRNA fragments [27]. This system yields products with high fidelity and no residual exogenous sequences [45]. However, it has poor adaptability for the circularization of ultra-long, large RNA fragments, as long-chain sequences are prone to misfolding, thereby hindering the circularization reaction [66]. Current optimization strategies mainly include the following:

(1) Using a computational “lock-and-key” design to achieve spontaneous proximity of RNA ends through sequence annealing, relying on T4 Rnl2 for efficient catalytic circularization, with a circularization efficiency exceeding 70% for 74–500 nt RNA. Among them, the efficiency for a 74 nt short sequence can reach 92%, and after optimization, the circularization efficiency for an 1100 nt sequence reaches 74%. The product does not require fine purification by Ribonuclease R (RNase R) or HPLC [45].
(2) Translational co-optimization: avoiding non-specific base pairing between the IRES and the downstream gene of interest, disrupting unfavorable secondary structures through site-directed mutagenesis, relieving inhibition of the IRES functional domain, and simultaneously achieving dual enhancement of circularization efficiency and protein translation efficiency [62].
(3) Splint-free self-assembly strategy: rationally designing linear RNA precursors using RNA secondary structure prediction, allowing the ends to spontaneously form a nicked structure and achieving scarless ligation without exogenous splints. The maximum circularization efficiency reaches 63%, and the IRES translation activity is fully restored after circularization, with translation efficiency being enhanced by up to 26-fold [66,67,68,69].

Though T4 Rnl2 enables scar-free circRNA generation, most studies remain at cellular antigen expression validation. Due to poor performance for long transcripts, few animal vaccination-challenge studies employ this system for full-length viral antigens [35].

### 4.3. LRC System

The LRC system requires no exogenous enzymes or intron elements. It relies on the spontaneous folding of RNA based on its own base complementarity and stem-loop structures to bring the ends together for self-ligation, representing a novel, simple circularization scaffold that can achieve one-pot synthesis. This system features strong template modularity, a streamlined preparation process, and a low-cost threshold; however, the circularization outcome is highly dependent on the intrinsic folding properties of the RNA, and complex viral antigen sequences are prone to forming competitive secondary structures that inhibit self-circularization [70]. Current mainstream optimization approaches include the following:

(1) Bioinformatics-assisted sequence design: utilizing RNA secondary structure simulation algorithms to perform site-directed optimization of the base composition in terminal complementary regions, weakening non-specific folding, strengthening directional terminal pairing capability, and enhancing sequence universality and circularization stability [71].
(2) Optimization of buffer systems and ionic environment: fine-tuning Mg^2+^ concentration, reaction temperature, and pH to modulate RNA folding kinetics, reducing the formation of incorrect conformations, and significantly improving the self-circularization efficiency of long-fragment LRC [72].
(3) Fusion design with functional elements: modular integration of IRES, spacer sequences, and the LRC scaffold, achieving the assembly of the circularization scaffold and translation functional elements in one step, thereby ensuring efficient self-circularization while safeguarding subsequent antigen translation activity and meeting the needs of integrated molecular design for circRNA vaccines [73].

LRC features simplified workflows yet exhibits strong sequence-dependent circularization. To date, no published viral circRNA vaccine animal immunization-challenge data using LRC are available, requiring further validation for in vivo vaccine use [70].

### 4.4. CIRC Self-Splicing Intron System

The CIRC self-splicing intron system is based on the natural self-splicing activity of unmodified, complete group I or group II introns, directly utilizing intact, unsplit intron sequences to catalyze RNA precursor circularization through two consecutive transesterification reactions. By leveraging the self-splicing activity of natural complete introns, the CIRC system achieves high-efficiency, broadly applicable, ultra-long, scarless, and easily purifiable circularization in a single-step co-transcription reaction [64]. However, its stability for large-scale production, sequence universality, and delivery compatibility still require sufficient validation. Optimization can be achieved through the following methods:

(1) Screening for highly efficient self-splicing introns and implementing structure-guided site-directed mutagenesis to enhance circularization efficiency and sequence adaptability [63].
(2) Fine-tuning parameters such as Mg^2+^ concentration, pH, and NTP ratios in the co-transcription reaction system to balance transcription and circularization kinetics, thereby suppressing non-specific by-products [22].
(3) Utilizing bioinformatics tools to predict the secondary structure of the fusion RNA, and introducing spacer sequences or synonymous mutations to disrupt competitive stem-loops, restoring the native intron conformation and ensuring circularization efficiency [74].

The emerging CIRC platform supports scar-free circularization of long transcripts and has achieved proof of concept in mouse immunization-challenge tests for an RSV vaccine. Nevertheless, public in vivo vaccine cases are limited, and efficacy data post-GMP scale-up remain absent [64].

**Table 2:** Comparison of technical characteristics of four main in vitro circularization methods for circular RNA.

Comparison Item	Permuted Intron–Exon	T4 RNA Ligase 2 Mediated Ligation	Linear RNA Self-Circularization	Complete Self-Splicing Intron for RNA Circularization	References
Circularization principle	Two-step transesterification self-splicing circularization of group I introns	Enzyme-catalyzed ligation of RNA 3′-OH and 5′-phosphate ends	Spontaneous folding of complementary sequences at RNA molecule ends	Based on the second-step splicing reaction of natural complete group I or group II introns	[27,64]
Template design features	Intron permutation and splitting, with the target gene inserted between exon regions (E1/E2)	Design linear RNA precursor, using terminal secondary structure self-annealing or a splint to bring the ends into proximity	Adopt a computationally optimized “lock-and-key” terminal complementary structure, bringing the 5′ and 3′ ends into spontaneous proximity through annealing	Use natural complete intron, without splitting, and insert the target gene inside the intron	[27,43,45,64]
Key conditions	GTP, Mg^2+^, high-temperature circularization	T4 Rnl2, ATP, mild conditions	Enzyme-free or with T4 Rnl2, mild conditions	Basic transcription buffer, mild conditions	[27,64]
Cyclization efficiency	87–95%	70–95%	70–73%	80–100%	[35,45,64,75]
Applicable RNA length	Long-sequence RNA	Small- to medium-length RNA	Tens to thousands of nt	Long-sequence RNA	[35,45,64]
Advantages	Wide application; relatively simple reaction conditions	Precise product; no exogenous sequences	Streamlined process; low cost	High efficiency; low immunogenicity	[40,64,67]
Limitations	Multiple steps; potential immunogenicity	Low circularization efficiency for large RNA fragments; splint assistance needed in some cases	Highly dependent on RNA folding properties and structural design precision	Relatively new technology; incomplete system validation	[40,66,76]
Main by-products	Uncircularized linear precursors, excised intron fragments, scar sequences, nicked circRNA, concatemers	Linear RNA precursors, splint-DNA residuals, mis-ligated concatemers, nicked circRNA	Misfolded concatemers, partial nicked circRNA, linear side-products	Excised intron debris, minor fraction of nicked circRNA	[12,35,64]
Vaccine development readiness	High; pre-clinical immunization-challenge studies in mice, non-human primates for SARS-CoV-2, monkeypox, and other viruses	Moderate–low; mostly cellular-level validation for short-to-medium antigens; scarce animal-challenge reports for full-length viral antigens	Low; only in vitro molecular and cellular data	Moderate; limited proof-of-concept mouse challenge data for viral vaccines; GMP-scale animal efficacy data still absent	[7,29,64,65,70]

### 4.5. Quantitative Comparison of Circularization Platforms and Linear mRNA for circRNA Vaccines

Linear mRNA adopts mature scalable workflows of in vitro transcription (IVT), capping and purification, with crude IVT yields of 2–5 g/L and 60–70% full-process recovery [77]. CircRNA adds an extra circularization step, increasing process complexity. All metrics originate from pre-clinical lab-scale tests; performance may vary greatly under GMP large-scale production [43].

Of four platforms, PIE (70–87%) suffers efficiency loss for sequences >1 kb and bears scar-sequence risks for animal immunization [35]. T4 Rnl2 (70–95%) works well only for short-to-medium transcripts, while splint-DNA impurities hinder scale-up [78]. LRC (70–73%) features simple operations yet is strongly sequence-dependent, with scarce in vivo vaccine evidence [40]. CIRC achieves 80–100% efficiency for long transcripts with minimal scar impurities and preliminary animal-vaccine validation, while lacking mature GMP scale-up experience [64].

Across platforms, purified circRNA delivers only 40–60% full-process recovery, which is lower than linear mRNA. CircRNA generates topology-specific impurities requiring orthogonal assays for batch release. Though free of expensive cap analogs, circRNA’s theoretical cost advantage is offset by reagent consumption and purification-related yield loss; its pre-clinical manufacturing cost exceeds optimized linear mRNA [43,77].

For viral circRNA vaccine development, CIRC holds the greatest comprehensive potential despite limited scale-up data. PIE and T4 Rnl2 fit short-antigen exploratory studies, whereas LRC demands further in vivo validation for vaccine use [64].

### 4.6. Critical Quality Attributes and Quality Control Status of circRNA Vaccines

CircRNA vaccines possess unique critical quality attributes distinct from linear mRNA, including closed-loop fraction, ligation-junction fidelity, nicked circRNA, dsRNA and various process residuals. No dedicated pharmacopeia is available so far [77]. Orthogonal assays such as RNase-R digestion, capillary electrophoresis and nanopore sequencing are adopted for characterization [71,77].

Each analytical method has inherent limitations: RNase-R cannot reliably separate intact circRNA from nicked species, while high-precision methods remain costly for routine batch release. Circularization workflows generate divergent impurity profiles, and in vivo dose–effect data for contaminants together with universal reference materials are lacking [78]. Establishing GMP-compatible quality control frameworks and safe impurity thresholds constitutes a prerequisite for the clinical translation of circRNA vaccines [40].

## 5. Molecular Design and Antiviral Immune Mechanisms of circRNA Vaccines

The core of target design for circRNA vaccines lies in selecting antigenic epitopes capable of inducing high-quality neutralizing antibodies and/or potent T-cell immunity and placing their coding sequences within an IRES-driven ORF. Current optimization focuses on improving translation efficiency and antigen expression levels (IRES selection, UTR/spacer sequences, impurity removal, chemical modification, etc.) and enhancing protective efficacy through combined antigen or fusion protein strategies.

### 5.1. Vaccine Target Design and Optimization

Target selection and antigen design are key determinants of immunogenicity and protective efficacy. The core strategy includes three aspects: first, priority screening of broadly conserved antigens/epitopes, such as selecting the receptor binding domain (RBD) for severe acute respiratory SARS-CoV-2 vaccines and constructing trimers to enhance immunogenicity [6], and adopting envelope domain III-Fc fusion protein (EDIII-Fc) dimers for Zika virus vaccines to induce stronger neutralizing antibody responses [79]; second, adopting combined antigen or multivalent design, where co-administration of Zika virus EDIII-Fc with non-structural protein 1 (NS1) achieves complete protection [79], and monkeypox vaccines using 2A peptide (2A)-mediated tandem co-expression of dual antigens enhance protective efficacy [65]; third, enhancing immunogenicity through structural optimization, such as introducing a tissue-type plasminogen activator (tPA) signal peptide to promote antigen secretion, or fusing a foldon domain to induce trimerization to mimic the native conformation [80], thereby further boosting the body’s immune response.

### 5.2. Strategies to Improve Translation Efficiency

Target-oriented circRNA vaccines use virus-specific antigens as the core to construct a basic “IRES-ORF” expression cassette. Through coordinated sequence optimization and delivery systems, antigens are stably and persistently expressed in antigen-presenting cells, inducing humoral and cellular immunity [46]. The core advantage is the long half-life conferred by the closed-loop structure of circRNA, with an immune stimulation duration far exceeding that of linear mRNA [80,81]. Improving the translation efficiency of circRNA is primarily achieved through four core strategies:

(1) Selecting and engineering potent IRES: Studies have shown that screening the enterovirus A (EV-A) IRES with excellent universality and truncating it to retain the core functional domain can increase translation efficiency by approximately 50%. Mutagenesis screening of the coxsackievirus B3 (CVB3) IRES yielded mutants such as A62G and U287A that could double protein yield [57]. Inserting eukaryotic translation initiation factor 4G (eIF4G) recruitment aptamers into specific loop regions of strong IRES or adopting dual IRES combinations can also significantly enhance translation [73,79].
(2) Optimizing sequences upstream and downstream of the IRES-ORF expression cassette: Introducing binding motifs for RNA-binding proteins such as polyA binding protein (PABP) and PolyC Binding Protein (PCBP) upstream and downstream of IRES-ORF promotes the formation of the translation initiation complex. Adding spacer sequences such as polyA50 between the IRES, coding region, and residual sequences can alleviate structural interference [22]. Adding the woodchuck hepatitis virus posttranscriptional regulatory element (WPRE) and a poly(A) stretch at the 3′ end can increase translation by more than 5-fold, and removing specific short sequences within WPRE can further restore expression by approximately 2-fold [73].
(3) Introducing epigenetic modifications and purification optimization: Supplementing N6-methyladenosine Triphosphate (N6-methyl-ATP) during IVT to introduce m^6^A modification can enhance cap-independent translation by recruiting YTH Domain Family Protein 3 (YTHDF3), while reducing immunogenicity and maintaining RNA stability. Thoroughly purifying circRNA to reduce linear impurities can prevent innate immune activation from interfering with translation [71].
(4) Employing circRNA technology to covalently attach a 7-methylguanosine (m7G) cap via a branched structure to form capped circular RNA, which can increase protein expression by approximately 7-fold compared to traditional IRES-circRNA: Non-covalent capping can enhance translation by over 50-fold and supports a rolling circle-like translation mode [82].

### 5.3. Future Design Directions for circRNA Vaccines

With advantages such as high stability, durable expression, and low immunogenicity, circRNA vaccines have become an important direction for next-generation nucleic acid vaccines. Future efforts can focus on AI-assisted design, virus-specific design, clinical translation, and cross-disciplinary applications to provide new strategies for combating emerging and re-emerging infectious diseases and viral infections.

(1) AI-assisted design: In the field of bioinformatics, deep learning models such as convolutional neural networks powered by AI can enhance the performance of antigen prediction, RNA structure modeling, and lipid nanoparticle delivery system optimization, surpassing traditional bioinformatics methods. Integrating AI-driven tools with conventional bioinformatics and experimental validation can elevate the level of vaccine development while ensuring computational efficiency and biological reliability [83].
(2) Virus-specific design: For pan-variant protection, Qu et al. designed a circRNA vaccine targeting the RBD region of the SARS-CoV-2 Delta variant that induced broadly neutralizing antibodies in the body, effectively neutralizing multiple SARS-CoV-2 variants including Omicron [6]. For viral infectious diseases in livestock and poultry, Liu et al. designed a circular mRNA-LNP vaccine with E2-TMD-mi3 self-assembling nanoparticles, which significantly enhanced specific immunity against classical swine fever virus compared to commercially available subunit vaccines [48]. Tian et al. prepared a circRNA nanovaccine encoding the sigma C protein coated with chitosan oligosaccharide-polyethyleneimine, which induced protective immunity against novel duck reovirus in ducks [47].
(3) Safety evaluation: Before newly developed circRNA vaccines can be officially launched on the market, a series of rigorous safety evaluations must be completed, which is a key prerequisite to ensure their widespread use. The four major hidden dangers—purification impurities of circRNA vaccine preparations, toxicity of the delivery vehicle, delayed side effects of long-term administration, and antibody-dependent enhancement—must be individually verified through a full suite of in vivo and in vitro safety tests. Currently, only preclinical basic safety screening can be completed, and a complete clinical safety evaluation system has not yet been established, which is the core bottleneck for the clinical application of circRNA vaccines against emerging infectious diseases [42].
(4) Combined immunotherapy: Liu et al. developed a dual-antigen circRNA vaccine based on a dimeric EDIII fused with the human IgG1 Fc fragment and the Zika virus non-structural protein NS1, which effectively protected mice against Zika virus infection without any observed antibody-dependent enhancement [79]. Yue et al. demonstrated that a trivalent circRNA vaccine effectively protected mice from infection by multiple influenza A H1N1, influenza A H3N2, and influenza B viruses [84]. Qu et al. prepared a circRNARBD-Delta vaccine that can be used as a booster after two doses of a Wuhan-Hu-1 or Delta-specific vaccine against SARS-CoV-2 [6].

## 6. Vaccine Delivery Systems

### 6.1. Oral, Intranasal, Aerosol, and Needle-Free Administration Routes

Needle-free administration can be broadly categorized into mucosal immunization and cutaneous immunization. The former refers to the delivery of drugs to the mucosa via oral, intranasal, spray, and other routes to induce mucosal immunity; the latter refers to the use of instantaneous high pressure to form a high-speed liquid jet that can penetrate the skin barrier without a needle, precisely delivering the drug to the dermis or subcutaneous tissue [85,86].

#### 6.1.1. Mucosal Immunization

Immune responses occurring on the mucosa, i.e., mucosal immunity, are an important component of the body’s immune system, consisting of immune components, immune cells, and antibodies [87]. A group of lymphoid tissues exists in the mucosa, known as mucosa-associated lymphoid tissue (MALT). MALT can be further subdivided into gut-associated lymphoid tissue, nasopharynx-associated lymphoid tissue, bronchus-associated lymphoid tissue, conjunctiva-associated lymphoid tissue, and vagina-associated lymphoid tissue, and different administration methods stimulate MALT at the corresponding sites [88]. MALT is composed of lymphoid follicles covered by microfold cells (M cells). M cells are responsible for taking up antigens from the lumen and presenting them to dendritic cells (DCs) and macrophages, where the antigens are processed and presented to lymphocytes, ultimately promoting B cell differentiation into plasma cells and the production of specific secretory immunoglobulin A (SIgA) and IgG, thereby generating immunity [62,89]. Measuring the titers and durability of IgA and IgG in vivo allows for a preliminary assessment of the vaccine’s immune efficacy [90]. Mucosal vaccines have significant advantages over traditional injectable vaccines: (1) they can be administered without needles, reducing pain; (2) spray and oral vaccines can greatly improve the efficiency of mass immunization campaigns; (3) the generation of memory cells by immunization is crucial for blocking transmission and preventing re-infection; and (4) they are amenable to chemical modification [91]. Despite these advantages, several challenges remain for mucosal vaccines: (1) how environmental factors modulate systemic and local immune responses; (2) how host factors affect vaccine efficacy; (3) safe and effective delivery systems and adjuvants; (4) how to establish broad and durable immune memory at mucosal sites; and (5) how to determine the correlates of mucosal immune protection [92].

#### 6.1.2. Cutaneous Immunization

Cutaneous immunization is primarily achieved through needle-free jet injection technology, a revolutionary transdermal drug delivery method with a development history of 75 years [85]. The structure of a needle-free jet injector can be simplified into a power source, a drug push rod, an injection chamber, and a nozzle. The principle of cutaneous immunization is largely the same as that of mucosal immunization, differing in the site and method of entry into the body: a high-speed liquid jet is formed by high pressure, delivering the drug subcutaneously to exert its effect. After delivery, the high-velocity particles dispersed in the tissue increase the interaction with immune cells, thereby enhancing the immune response elicited by the vaccine and generating stronger immunity [93]. Furthermore, for some diseases, needle-free jet injection technology achieves essentially the same immunity as needle-based technology using a lower drug dose [94]. This technology can fundamentally eliminate needle phobia, the risk of needlestick injuries, and the infection problems caused by needle sharing in actual production [86,95]. Currently, needle-free jet injection products that are marketed or under development for vaccines include: a spring-powered needle-free jet injection system for mRNA vaccination against SARS-CoV-2 [93]; a compressed carbon dioxide-powered needle-free jet injection system for DNA vaccination against HIV [88]; and a spring-powered needle-free jet injection system for DNA vaccination against Venezuelan equine encephalitis [96]. In these products, needle-free technology has facilitated vaccine delivery and demonstrated favorable immunogenicity.

### 6.2. Delivery Vectors

Representative RNA delivery vectors include lipid nanoparticles, exosomes, bacteriophages, and virus-like particles, which differ significantly in cargo capacity, delivery efficiency, and route of administration (Table 3).

#### 6.2.1. Advances in LNP Research

LNPs are a highly promising delivery vehicle for nucleic acid therapeutics and vaccines, playing a crucial role in the delivery of mRNA [97]. During antigen presentation, LNPs can safely and efficiently carry mRNA vaccines across multiple biological barriers to target cells, ensuring that the mRNA vaccine reaches the target cells to function, activate specific T cells, and induce an immune response [98,99]. Currently, third-generation LNP delivery systems, which include the ionizable cationic lipid ALC-0315 and the amino cationic lipid SM-102, have high transfection efficiency for long-chain mRNA in vivo and have been used in the production of COVID-19 mRNA vaccines [100].

Although LNPs have enabled substantial advances in RNA vaccine delivery, challenges remain with respect to endosomal escape efficiency, tissue-specific biodistribution, and formulation-dependent reactogenicity. First, the endosomal escape efficiency of LNP–RNA complexes is generally low [101]. Moreover, the underlying mechanisms remain unclear, and some findings even appear contradictory. Nevertheless, it has been suggested that elucidating the escape sites, together with the development of turn-on quantitative assays and high-resolution microscopy techniques, could help address this issue by informing the design of smart delivery strategies and enabling the prediction of potent ionizable lipid structures [102]. Second, LNPs predominantly target the liver, which limits their therapeutic efficacy in other tissues and organs. This is largely attributable to the rapid formation of a plasma protein corona on the LNP surface upon intravenous administration; these adsorbed proteins are capable of specifically binding to the low-density lipoprotein receptor on hepatocytes, thereby directing LNPs to these cells and facilitating subsequent endocytic uptake. In addition, the relatively slow blood flow, high vascular permeability, and abundance of macrophages in the liver may further contribute to the preferential accumulation of LNPs in this organ [103]. Finally, in terms of formulation-dependent reactogenicity, studies have found that cholesterol-based LNPs are more suitable for strong immunity. In contrast, the introduction of β-sitosterol into LNPs may reduce the pro-inflammatory response, providing a direction for developing applications such as tolerogenic vaccines or non-vaccine RNA therapeutics [104]. Although LNPs have brought significant advances to mRNA delivery, it has been reported that most LNP-encapsulated mRNA vaccines exhibit extremely strong mRNA expression in the liver after intravenous or intramuscular administration, and even reverse transcription into DNA, causing liver damage [105,106].

#### 6.2.2. Other Delivery Vectors

T4 bacteriophage as a delivery vector has the following advantages: first, the particle size of the bacteriophage is typically 20 to 200 nanometers, facilitating its efficient transport in animals. In addition, its particle structure, which is easily phagocytosed, and its adaptability to multiple endocytic mechanisms make it more readily internalized by cells, thereby inducing an immune response more quickly; second, the capsid protein of T4 bacteriophage has a repetitive and symmetrical structure, making it easier to interact and cross-link with extracellular pattern recognition receptors, triggering inflammatory signaling; third, the bacteriophage contains genomic DNA and RNA, which can be detected by multiple pattern recognition receptors; fourth, the high copy number of capsid proteins allows for the modular integration of antigens into the bacteriophage particle; fifth, the bacteriophage surface may carry small amounts of bacterial lipopolysaccharide [107]. In addition, some other delivery vectors under investigation, such as Bacillus subtilis, attenuated Salmonella cholerae, and polydopamine, have shown advantages and prospects in their respective fields [108,109,110].

**Table 3:** Comparison of the efficacy of representative RNA delivery vectors.

Delivery Vector	Key Component	Maximum Cargo Capacity and Delivery Efficiency	Route of Administration	Advantage	Limitation	References
Lipid nanoparticle	Ionizable lipids, cholesterol, helper lipids, and PEGylated lipids	mRNA encapsulation efficiency >35%, endosomal escape rate 25–80%	Intramuscular injection	High clinical maturity; relatively high delivery efficiency; strong applicability	High cytotoxicity; overly broad and non-specific delivery targets; large variability in efficacy for different RNA types	[111,112,113]
Exosome	Lipid bilayer structure, surface-specific marker proteins	mRNA encapsulation efficiency ~90%, high delivery efficiency	Intranasal, intravenous injection	High biocompatibility; low immunogenicity; natural cell targeting	Lack of purification methods; low loading efficiency for large RNA molecules	[114,115,116]
Bacteriophage T4	Capsid and nucleic acid	171 kb, up to ~100% for the natural host E. coli, but low delivery efficiency to other cells	Intranasal administration	Extremely strong loading capacity; non-infectious to animal cells; low cost; rapid production	Low efficiency of entry into animal cells; complicated genetic engineering procedures; potential immunogenicity	[117,118,119,120,121]
Virus-like particles	Capsid; enveloped or non-enveloped	mRNA of 500 bp to 10 kb, delivery efficiency ranging from 15% in natural killer cells to 65% in T cells	Intravenous injection, intramuscular injection	Lack infectivity and replication capacity; high safety; uniform size, easy aggregation	Limited targeting; difficult high-purity, low-cost industrial production; limited in vivo data; high variability in delivery efficiency across different cells	[122,123,124,125,126,127,128,129]

Overall, lipid nanoparticles have the highest clinical maturity, with mRNA encapsulation efficiency exceeding 35% and a high upper limit of endosomal escape rate, but they possess certain cytotoxicity and reactogenicity [111,112,113]. Exosomes have high biocompatibility and natural targeting ability; however, their purification methods and loading efficiency for large RNA molecules are the main bottlenecks [114,115,116]. Although T4 bacteriophage has an extremely strong loading capacity (171 kb) and low cost, its delivery efficiency to animal cells is low, limiting its direct use in mammalian systems [117,118,119,120,121]. Virus-like particles have high safety, but their delivery efficiency varies greatly across different cells, and large-scale production is challenging [122,123,124,125,126,127,128,129].

Based on the comprehensive comparison in Table 3, if rapid clinical translation is the goal, lipid nanoparticles are the current preferred choice; if low immunogenicity and the ability to cross physiological barriers are pursued, exosomes have greater promise. Bacteriophages and virus-like particles require further engineering to improve targeted delivery efficiency.

### 6.3. Strategies to Improve Targeted Delivery Efficiency

The delivery of conventional antigens and adjuvants to target organs typically encounters two major obstacles—anatomical barriers and the drainage function of the lymphatic system—thereby affecting delivery efficiency [130].

Currently, strategies to improve targeted delivery efficiency can be approached through the design optimization of nanomaterials, biomimetic nanomedicines, and controlled drug release. By engineering the surface of drug-carrying nanomaterials and modifying characteristics such as surface charge, particle size, and shape, precise navigation and efficient penetration of biological barriers can be achieved [131,132]. Furthermore, modifying nanoparticles with various ligands capable of specifically targeting multiple components of the tumor microenvironment can achieve precise drug release at the right time and place, reducing off-target effects [133]. Finally, as biomimetic nanomedicines mimic natural biological structures, they enhance immune evasion and active targeting capabilities [134].

### 6.4. Practical Implementation of Needle-Free Technology

Traditional needle injection is currently the mainstream method for vaccinating livestock, poultry, and humans, but it has many insurmountable limitations in large-scale immunization scenarios at the grassroots level, severely constraining the efficiency of viral disease prevention and control. In the livestock and poultry industry, needlestick injuries have become the primary occupational exposure risk for veterinarians. An epidemiological survey of 794 swine veterinarians showed that 73% of respondents had experienced needlestick injuries of varying severity [135]; simultaneously, the stress response induced by needle injection in animals can significantly reduce vaccine immune efficacy [136], and the reuse of needles also poses a potential risk of cross-contamination [95,137]. In addition, injection site lesions cause direct meat economic losses, with data indicating that approximately 7% of beef hindquarters and 15% of dairy cow hindquarters in the United States have injection site damage [138], and the use of large quantities of disposable needles also brings the additional burden of medical waste sorting and disposal [139].

Needle-free drug delivery technology, through mucosal immunization or high-pressure jet delivery, can fundamentally address the inherent drawbacks of needle injection mentioned above, aligning closely with the grassroots application needs of circRNA vaccines. This technology enables precise vaccine delivery without needles, not only avoiding occupational exposure and cross-contamination but also significantly improving the efficiency of large-scale centralized immunization. It can simultaneously induce dual mucosal and systemic immune responses, which better meets the prevention and control needs of viral diseases transmitted via respiratory and digestive tracts [140]. However, the combined application of needle-free technology and circRNA vaccines still faces multiple bottlenecks and has not yet achieved large-scale clinical and field implementation.

At the equipment level, there are challenges of cost and standardization: the procurement and maintenance costs of needle-free jet injection devices are significantly higher than those of traditional syringes, and the operational procedures vary greatly among products from different manufacturers, requiring grassroots operators to undergo specialized training to use them correctly. The issue of formulation stability is particularly prominent: the instantaneous high shear forces generated during needle-free jet injection may disrupt the structural integrity of the LNP-circRNA complex, leading to RNA degradation and reduced transfection efficiency; simultaneously, the strong local inflammatory response triggered by needle-free delivery accelerates the clearance of antigen-expressing cells, directly undermining the core advantage of long-lasting antigen expression of circRNA vaccines [75]. Finally, the related research foundation is relatively weak: there is currently a paucity of studies on the kinetic properties of circRNA and LNP carriers under needle-free jet injection conditions and a lack of parameter optimization data for different animal species and injection sites, which cannot provide scientific support for formulation development and equipment adaptation. Furthermore, certain doubts remain among grassroots farmers and the public regarding the immune effectiveness of needle-free injection technology, to some extent hindering its promotion and application [86,141,142].

## 7. Application of circRNA Vaccines in Viral Infectious Diseases

circRNA has attracted significant attention due to its unique structure and functions, making it a strong candidate for next-generation vaccines. Owing to its non-canonical translation mechanism and its structure lacking 5′ and 3′ ends, circRNA vaccines have demonstrated durable antigen expression capability in preventing infectious diseases and elicit a higher proportion of neutralizing antibodies at the same dose compared to mRNA vaccines [55].

### 7.1. Advantages of circRNA Vaccines

As outlined in the Introduction, the enhanced biological stability of circRNA supports sustained antigen expression in vivo. Zhang et al. used intron–exon elements to generate circRNA through in vitro back-splicing of a linear template, then packaged and delivered it with an LNP to create a vaccine against herpes simplex virus type 2. Challenge experiments in mice showed that the circRNA vaccine successfully induced significant humoral and cellular immune responses against HSV-2 in mice, and compared with conventional mRNA vaccines, the circRNA vaccine triggered a more durable immune response; in post-immunization-challenge experiments, all mice survived with low viral loads, demonstrating its effective protection against viral infection and attack [143]. Wu et al. developed a bivalent circRNA vaccine primarily targeting the major antigens B6 and M1 of monkeypox virus. Experiments showed that this vaccine induced efficient specific antibodies and T-cell responses against B6 and M1. In addition, the vaccine provided sufficient cross-protection to mice challenged with a lethal dose of vaccinia virus [144].

### 7.2. Current Status and Gaps in circRNA Vaccine Research

Several recent studies (as shown in Table 4 below) have explored the potential application of LNP-delivered circRNA vaccines against multiple viral pathogens. Although these studies have yielded generally positive results, all findings are derived from animal models without clinical validation. The delivery vehicle remains limited to LNPs, with no exploration of alternative delivery systems. Furthermore, none of these candidates have been advanced to commercial manufacturing, and critical practical aspects—including broad-spectrum efficacy, cost-effectiveness, and storage and transportation feasibility—remain unaddressed, indicating a considerable gap before commercialization can be achieved.

## 8. Future Directions

With the long-lasting protein expression and low immunogenicity conferred by their closed-loop structure, circRNA vaccines have become a cutting-edge platform for nucleic acid vaccines, yet their clinical translation and industrialization for viral disease prevention and control still face multiple challenges [55]. The current core technical difficulties are concentrated in three areas: circularization efficiency, cost, and delivery. First is the bottleneck in the circularization efficiency of long fragment viral antigen genes: most viral antigen genes are >1 kb, and the efficiency of the PIE strategy for long sequences drops sharply from >90% to less than 30% [40,43]; for sequences >3 kb, secondary structure interference of the RNA precursor leads to circularization failure [145]; moreover, after changing the sequence, re-optimization is required, making “plug-and-play” impossible [43,77,146]. Developing sequence-universal circularization scaffolds or using AI to eliminate secondary structures is a key direction [40,145]. Second is production cost control: existing processes rely on RNase R digestion and HPLC fine purification, which struggle to meet the extremely low-cost requirements of livestock and poultry vaccines [1,15,76]; it is necessary to improve circularization efficiency to >95% in order to replace fine purification with TFF [75], utilize rolling circle translation to amplify protein output [147,148], and repurpose mRNA production lines to compress costs [149]. Third is the challenge of grassroots-adapted delivery technology: the shear force of needle-free delivery causes LNP destabilization, and dermal nucleases degrade naked RNA; although circRNA lacks free ends, its enzymatic degradation resistance still needs verification [150]; the local inflammation induced by needle-free delivery may accelerate the clearance of expressing cells, diluting the long-acting advantage; coupled with the difficulty of co-adapting formulation and equipment, parameters for different animal species cannot be directly applied, requiring the establishment of a “formulation mechanics–skin transport–immune effect” framework and an animal species-specific parameter database.

Looking ahead, industrialization should focus on two core scenarios: first, routine prevention and control of viral diseases in livestock and poultry, leveraging the characteristics of persistent expression, rapid sequence updating, and lyophilization to break free from the cold chain and to create a next-generation vaccine platform suitable for grassroots use [71]; second, emergency vaccine stockpiling for emerging and re-emerging zoonotic diseases, relying on ambient temperature stability and rapid design advantages to establish a modular circRNA vaccine backbone library coupled with lyophilization processes, allowing construction and production to be completed within weeks after the emergence of a new pathogen, as validated in models such as Nipah virus and Zika virus [42,144]. Industrial implementation also requires the establishment of dedicated quality control standards for circular topology to achieve regulatory adaptation [76,151].

## Figures and Tables

**Table 4:** Research overview of representative circRNA vaccines.

Vaccine	Target Antigen	Delivery Vector	Immunological Efficacy	Practical Production Status	References
circRNA^RBD^	Encodes the RBD of the SARS-CoV-2 spike protein	LNP	Induces sustained humoral immune responses and high titers of neutralizing antibodies	Not yet applied in practical production	[6]
circRNA^B6M1^	Envelope antigen B6 (extracellular virion) and matrix antigen M1 (intracellular mature virion) of monkeypox virus (MPXV)	LNP	Effectively induces neutralizing antibodies against MPXV and exhibits cross-reactivity against vaccinia virus (VACV)	Not yet applied in practical production	[144]
EDIII-Fc circRNA EDIII-Fd circRNA EDIII circRNA	EDIII-Fc: fusion protein of Zika virus envelope domain III (EDIII) and human IgG1 Fc fragmentNS1: non-structural protein 1 of Zika virus	LNP	A single vaccination confers effective protection; the immunogenicity of EDIII-Fc circRNA is superior to that of EDIII-Fd or EDIII circRNA	Not yet applied in practical production	[79]
pUC57-circ-gD mRNA	Extracellular domain of glycoprotein D (gD) from herpes simplex virus type 2 (HSV-2)	LNP	Delivers durable, effective and safe immunogenicity	Not yet applied in practical production	[143]

## Data Availability

No new data were created or analyzed in this study. Data sharing is not applicable to this article.

## Abbreviations

The following abbreviations are used in this manuscript:

2A	2A peptide
5′-UTR	5′ untranslated region
3′-UTR	3′ untranslated region
APCs	Antigen-presenting cells
cDNA	Complementary DNA
CD4^+^	CD4-positive
CD8^+^	CD8-positive
ceRNA	Competing endogenous RNA
circRNA	Circular RNA
CIRC	Complete self-splicing intron
CVB3	Coxsackievirus B3
DCs	Dendritic cells
EDIII-Fc	Envelope domain III-Fc fusion protein
eIF4G	Eukaryotic translation initiation factor 4G
EV-A	Enterovirus A
HPLC	High-performance liquid chromatography
HSV-2	Herpes simplex virus type 2
HTNV	Hantaan virus
IgG	Immunoglobulin G
IRES	Internal ribosome entry site
IRF3	Interferon regulatory factor 3
IVT	In vitro transcription
LNP	Lipid nanoparticle
LRC	Linear RNA self-circularization
M cells	Microfold cells
MALT	Mucosa-associated lymphoid tissue
MAVS	Mitochondrial antiviral signaling protein
MDA5	Melanoma differentiation-associated gene 5
m^6^A	N^6^-methyladenosine
m7G	7-methylguanosine
MHC-I	Major histocompatibility complex class I
MHC-II	Major histocompatibility complex class II
miRNA	MicroRNA
N6-methyl-ATP	N6-methyladenosine triphosphate
NF-κB	Nuclear factor kappa-light-chain-enhancer of activated B cells
NLRs	NOD-like receptors
NS1	Non-structural protein 1
nsP1-nsP4	Non-structural proteins 1–4
NTP	Nucleoside triphosphate
ORF	Open reading frame
PABP	PolyA binding protein
PCBP	PolyC Binding Protein
PEG	Polyethylene glycol
PIE	Permuted intron–exon
PKR	Protein kinase R
poly(A)	Polyadenosine tail
RdRP	RNA-dependent RNA polymerase
RBD	Receptor binding domain
RIG-I	Retinoic acid-inducible gene I
RNase R	Ribonuclease R
saRNA	Self-amplifying RNA
SIgA	Secretory immunoglobulin A
ssRNA	Single-stranded RNA
T4 Rnl2	T4 RNA ligase 2
T4Td	T4 bacteriophage thymidylate synthase gene intron
TFF	Tangential flow filtration
TLR3	Toll-like receptor 3
TLR7/8	Toll-like receptor 7/8
tPA	Tissue-type plasminogen activator
WPRE	Woodchuck hepatitis virus posttranscriptional regulatory element
YTHDF3	YTH Domain Family Protein 3
